# Clinical Significance of Neutrophil-to-Lymphocyte Ratio and Lymphocyte-to-Monocyte Ratio in Oral Squamous Cell Carcinoma

**DOI:** 10.3390/diagnostics16111662

**Published:** 2026-05-28

**Authors:** Alina Ormenisan, Cecilia Petrovan, Adina Simona Coșarcă, Mihai Vlad Golu, Gabriela Felicia Beresescu, Mădălina Conțiu, Andreea Păcurar, Despina Luciana Bereczki-Temistocle

**Affiliations:** 1 Department of Oral and Maxillo-Facial Surgery, George Emil Palade University of Medicine, Pharmacy, Science, and Technology of Targu Mureș, 540139 Targu Mureș, Romania; alina.ormenisan@umfst.ro (A.O.); cecilia.petrovan@umfst.ro (C.P.); vlad.golu@umfst.ro (M.V.G.); despina.bereczki-temistocle@umfst.ro (D.L.B.-T.); 2 Department of Tooth and Dental Arch Morphology, George Emil Palade University of Medicine, Pharmacy, Science, and Technology of Targu Mureș, 540139 Targu Mureș, Romania; felicia.beresescu@umfst.ro; 3 Targu Mureș Emergency County Hospital, 540136 Targu Mureș, Romania; contiu.madalina@gmail.com; 4 Private Dental Practice, 540003 Targu Mureș, Romania; andreea.pacurar@icloud.com

**Keywords:** oral squamous cell carcinoma, inflammation, neutrophil-to-lymphocyte ratio, lymphocyte-to-monocyte ratio

## Abstract

**Background/Objectives**: Oral squamous cell carcinoma (OSCC) remains a significant global health concern, characterized by high morbidity and mortality, particularly in advanced stages. Increasing evidence suggests that systemic inflammation plays a critical role in tumor progression and patient outcomes. This retrospective cohort study aimed to evaluate the prognostic value of systemic inflammatory markers—specifically the neutrophil-to-lymphocyte ratio (NLR) and lymphocyte-to-monocyte ratio (LMR)—in correlation with histopathological parameters in patients with OSCC. **Methods**: A total of 157 patients diagnosed with histopathologically confirmed OSCC between January 2019 and December 2024 were included. Clinical, laboratory, and histopathological data were analyzed, including lymphovascular and neural invasion and worst pattern of invasion (WPOI). Statistical analyses, including ROC curve analysis, Kaplan–Meier survival analysis, and Cox regression, were performed to determine the prognostic significance of NLR and LMR. **Results**: The cohort demonstrated a predominance of male patients (81.5%), with the highest incidence observed in the 61–70 age group. WPOI showed a statistically significant association with survival outcomes (*p* = 0.003), with type 5 invasion correlating with increased mortality. ROC analysis identified optimal cutoff values of 2.88 for NLR and 1.87 for LMR. Elevated NLR and decreased LMR were significantly associated with poorer survival (*p* = 0.0001). Kaplan–Meier analysis confirmed reduced overall survival in patients with NLR ≥ 2.88 and improved survival in those with LMR ≥ 1.87. Cox regression analysis identified NLR as an independent predictor of mortality (HR = 2.05, *p* = 0.025), while LMR demonstrated a non-significant protective trend. **Conclusions**: These findings support the prognostic relevance of systemic inflammatory markers in OSCC. Unlike many previous studies evaluating inflammatory biomarkers alone, the present study additionally explored the relationship between NLR/LMR and histopathological invasion patterns, particularly WPOI, highlighting the potential interaction between systemic inflammation and tumor aggressiveness in OSCC. Further prospective, multicenter studies are required to validate these results and establish standardized clinical thresholds.

## 1. Introduction

Oral squamous cell carcinoma (OSCC) is a malignant neoplasm arising from the stratified squamous epithelium of the oral mucosa and represents one of the most common cancers of the head and neck region. Despite advances in surgical techniques, radiotherapy, and chemotherapy, OSCC continues to be associated with significant morbidity and mortality. Global incidence remains high, with nearly 400,000 new cases reported annually, while long-term survival rates remain critically low [1,2].

The development and progression of OSCC are influenced by a complex interplay of genetic susceptibility and environmental risk factors, including tobacco use, alcohol consumption, and viral infections. In addition to these factors, increasing evidence highlights the critical role of systemic inflammation in tumor progression and patient prognosis. In this context, easily accessible hematological markers have gained attention as potential prognostic tools. Among these, the neutrophil-to-lymphocyte ratio (NLR) and lymphocyte-to-monocyte ratio (LMR) reflect the balance between inflammatory response and immune surveillance and have been associated with clinical outcomes in various malignancies [2,3].

Histopathological parameters also play a crucial role in determining tumor behavior and prognosis. Features such as perineural invasion and worst pattern invasion (WPOI) have been shown to correlate with tumor aggressiveness, metastatic potential, and survival outcomes. However, the integration of systemic inflammatory markers with histopathological characteristics remains insufficiently explored, particularly in routine clinical practice [3].

Histopathological characteristics such as lymphovascular invasion (LVI), perineural invasion (PNI), and worst pattern of invasion (WPOI) are well-established indicators of tumor aggressiveness in oral squamous cell carcinoma (OSCC). These features reflect the local invasive capacity of the tumor and are associated with recurrence, nodal metastasis, and reduced survival. However, despite their prognostic relevance, they do not fully explain the heterogeneity in clinical outcomes among patients with similar pathological stages.

Increasing evidence suggests that systemic inflammation also contributes significantly to tumor progression and immune evasion. Hematological inflammatory markers, particularly the neutrophil-to-lymphocyte ratio (NLR) and lymphocyte-to-monocyte ratio (LMR), have emerged as accessible prognostic biomarkers in several malignancies, including OSCC. Elevated NLR and reduced LMR are thought to reflect a pro-tumor inflammatory microenvironment associated with impaired anti-tumor immunity.

Although both histopathological parameters and inflammatory biomarkers have individually demonstrated prognostic value, their relationship and potential complementary role remain insufficiently investigated. Therefore, the present study aimed to evaluate the prognostic significance of NLR and LMR in OSCC and to determine whether these systemic inflammatory markers provide additional prognostic information beyond conventional histopathological features such as LVI, PNI, and WPOI.

## 2. Materials and Methods

### 2.1. Study Design and Patient Population

This retrospective observational cohort study included patients diagnosed with oral squamous cell carcinoma (OSCC) who were admitted to the Department of Oral and Maxillofacial Surgery at the Târgu Mureș Emergency County Hospital between January 2019 and December 2024.

A total of 157 patients with histopathologically confirmed intraoral SCC were included in the study.

Ethical approval for the study was obtained from the institutional review board (No. 2773/26 January 2024). Because of the retrospective design of the study, patient data were collected from existing medical records. All patients had signed a general informed consent form at hospital admission, allowing the anonymized use of clinical and pathological data for research purposes. Patient confidentiality was preserved throughout the study in accordance with institutional regulations and the Declaration of Helsinki.

### 2.2. Inclusion and Exclusion Criteria

Patients were eligible for inclusion if they were older than 18 years, had a histopathologically confirmed diagnosis of intraoral squamous cell carcinoma (SCC), and had available preoperative blood test results.

Patients were excluded if they had incomplete medical records, lacked a confirmed histopathological diagnosis, were younger than 18 years, or had a history of extraoral malignancies.

Patients who received neoadjuvant radiotherapy, chemotherapy, immunotherapy, or biological therapy before surgery were excluded from the study to avoid treatment-related alterations of inflammatory markers.

### 2.3. Data Collection

Clinical, laboratory, and histopathological data were retrospectively collected from patient medical records. The analyzed variables included demographic characteristics (age and sex), tumor location, treatment details, and survival status. The follow-up period was between 8 and 72 months. Follow-up data were obtained by telephone or medical records review.

All included patients underwent primary surgical treatment for OSCC.

Histopathological parameters evaluated in this study included surgical margins, lymphovascular invasion, perineural invasion, and worst pattern of invasion (WPOI). Lymphovascular invasion (LVI) and perineural invasion (PNI) were assessed on routine hematoxylin–eosin-stained sections by experienced pathologists and recorded as binary categorical variables (present/absent). LVI was defined as the presence of tumor cells within endothelial-lined lymphatic or vascular spaces, while PNI was defined as tumor infiltration in, around, or through a nerve sheath. According to the Brandwein–Gensler grading system, WPOI can be classified into 5 subtypes:

- Type 1: broad pushing borders;
- Type 2: finger-like pushing invasion;
- Type 3: large tumor islands (>15 cells);
- Type 4: small tumor islands (≤15 cells);
- Type 5: tumor satellites located ≥1 mm from the main tumor or extratumoral perineural/lymphovascular invasion.

For additional statistical analysis, WPOI types 1–4 were grouped together and compared with WPOI type 5 because type 5 is generally considered the most aggressive invasion pattern.

Systemic inflammatory markers were calculated using preoperative blood test results, specifically the neutrophil-to-lymphocyte ratio (NLR) and lymphocyte-to-monocyte ratio (LMR).

### 2.4. Statistical Analysis

Statistical analysis was performed using SPSS version 23.0 (IBM Corp., Armonk, NY, USA) and MedCalc software (Version 22). Data distribution was assessed using the Kolmogorov–Smirnov test. Continuous variables were analyzed using Student’s *t*-test for normally distributed data and the Mann–Whitney U test for non-normally distributed data.

Associations between categorical variables were evaluated using the chi-square test. For contingency tables, the chi-square test was applied when expected frequencies were greater than 10, the chi-square test with Yates’ correction was used for expected frequencies between 5 and 10, and Fisher’s exact test was applied when expected frequencies were less than 5.

Overall survival (OS) was defined as the interval between the date of surgery and death or last follow-up. Kaplan–Meier survival analysis with log-rank testing was used to compare survival according to inflammatory marker cutoffs. Cox proportional hazards regression analysis was performed to evaluate whether NLR and LMR independently predicted mortality risk. Histopathological variables and inflammatory markers were also compared using univariate analyses to explore potential associations between systemic inflammation and tumor invasion characteristics.

Receiver operating characteristic (ROC) curve analysis was performed to determine the optimal cutoff values for NLR and LMR.

For all analyses, Pearson correlation coefficients (r) and two-tailed *p* ≤ 0.05 represented statistical significance (95% confidence interval).

## 3. Results

Of the 157 patients included in the study, the majority were male (81.5%), as shown in Table 1.

Patients were stratified into age groups (<50, 51–60, 61–70, 71–80, >80 years). The highest proportion was observed in the 61–70 age group (35%), followed by the 51–60 group (25.5%) (Table 2).

The relationship between histopathological invasion patterns (vascular, perineural, and lymphatic) and survival was analyzed. Although perineural invasion appeared more frequent among deceased patients, statistical analysis did not demonstrate a significant association (*p* = 0.53) (Table 3).

In contrast, the worst pattern of invasion (WPOI) showed a statistically significant association with survival outcomes (*p* = 0.003). WPOI type 5, characterized by satellite tumor clusters located more than 1 mm from the primary lesion and often associated with perineural or lymphovascular invasion, was strongly correlated with poor prognosis and increased mortality (Table 4).

**Figure 1:**
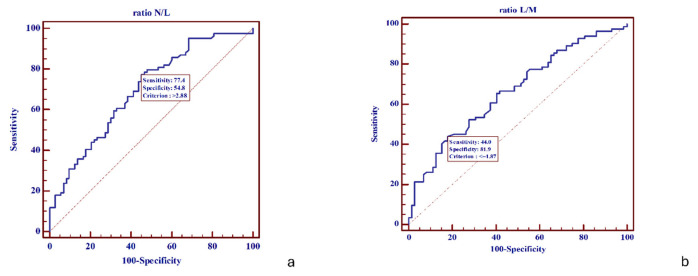
(**a**) ROC curve for N/L ratio, cutoff value of 2.88; (**b**) ROC curve for L/M ratio, cutoff value of 1.87.

Inflammatory markers were also evaluated. ROC analysis identified a cutoff value of 2.88 for NLR and 1.87 for LMR. Elevated NLR values above the cutoff were associated with poorer survival outcomes, while decreased LMR values were similarly linked to unfavorable prognosis (Figure 1).

Statistical testing (Mann–Whitney U test) confirmed that both NLR and LMR were significantly associated with negative patient outcomes (*p* = 0.0001), supporting their role as prognostic indicators (Table 5).

### Survival Analysis

**Figure 2:**
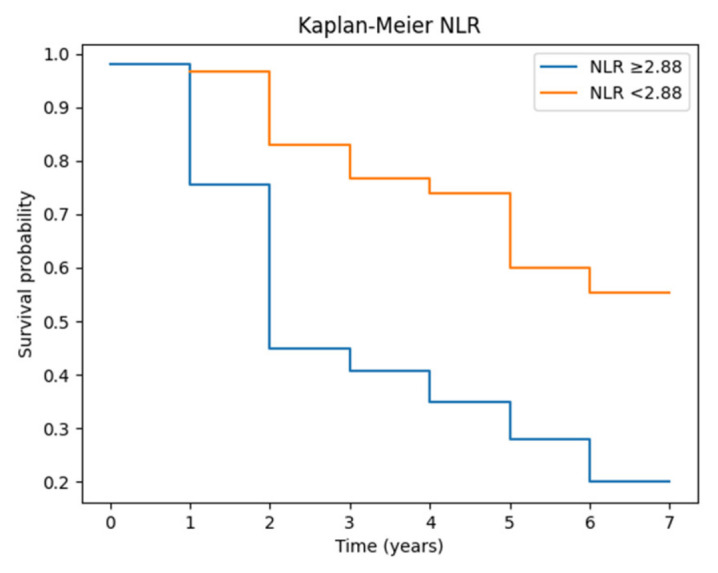
Survival analysis based on NLR stratification.

Kaplan–Meier survival analysis demonstrated significant differences in overall survival based on systemic inflammatory marker stratification (Figure 2).

Patients with an NLR ≥ 2.88 exhibited significantly reduced overall survival compared with those with an NLR < 2.88 (log-rank *p* = 0.0001).

**Figure 3:**
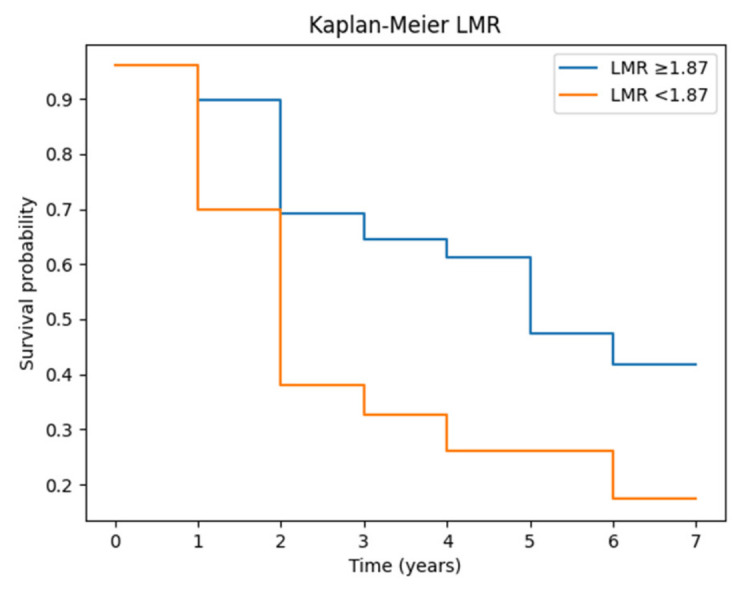
Survival analysis based on LMR.

Similarly, patients with an LMR ≥ 1.87 showed significantly improved survival compared with those with an LMR < 1.87 (log-rank *p* = 0.0004) (Figure 3).

Cox regression analysis demonstrated that elevated NLR was associated with increased mortality risk (HR = 2.05, *p* = 0.0250), while LMR showed a protective trend (HR = 0.67, *p* = 0.1183) (Table 6).

**Table 6:** Cox regression for NLR and LMR.

Variable	HR	*p*-Value	95% CI
N/L	2.05	0.0250	1.10–3.82
L/M	0.67	0.1183	0.35–1.28

Additionally, a combined prognostic score was constructed using the following factors: NLR > 2.88, LMR < 1.87, presence of perineural invasion, and WPOI 4–5. Each factor was assigned 1 point. Patients were stratified into three groups: low risk (0–1 points), intermediate risk (2 points), and high risk (3–4 points).

**Figure 4:**
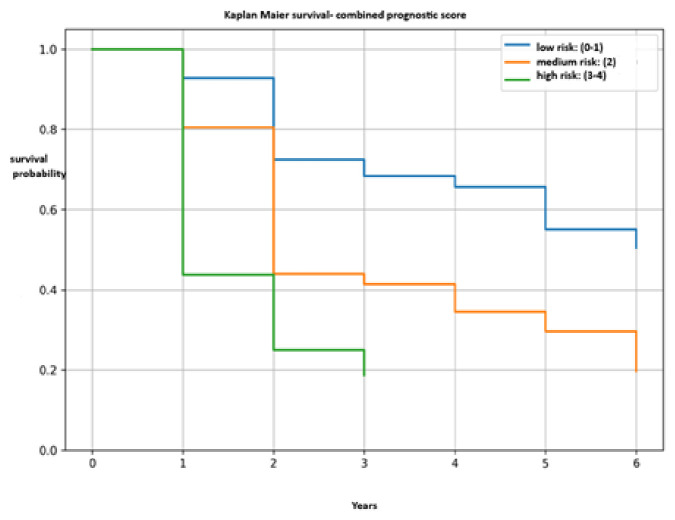
Kaplan–Meier combined prognostic score for survival analysis.

Kaplan–Meier analysis demonstrated significant differences in survival among the risk groups, with patients in the high-risk group showing the poorest survival compared to the low- and intermediate-risk groups (log-rank *p* < 0.001) (Figure 4).

In the Cox regression analysis, NLR >2.88 was associated with an increased risk of mortality (HR ≈ 2.4), whereas a higher LMR demonstrated a protective effect on survival. In addition, perineural invasion (HR ≈ 2.1) and WPOI 4–5 (HR ≈ 2.8) were associated with an unfavorable prognosis (Table 7). Patients with a high prognostic score exhibited significantly reduced survival.

**Table 7:** Cox regression analysis for multiple parameters: NLR, LMR, perineural invasion, and WPOI.

Variable	Group	HR	95% CI	*p* Log-Rank
**NLR**	>2.88 vs. ≤2.88	2.4	1.2–4.9	<0.001
**LMR**	<1.87 vs. ≥1.87	1.9	1.1–3.0	0.0002
**Perineural invasion**	present vs. absent	2.1	1.2–3.7	0.01
**WPOI**	4–5 vs. 1–3	2.8	1.5–5.3	0.003
**Combined score**	High vs. low risk	3.5	1.8–6.7	<0.001

**Figure 5:**
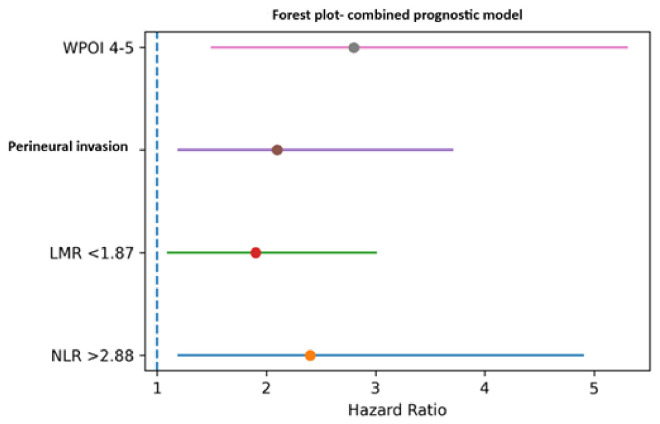
Forest plot of the combined prognostic model.

The association between high WPOI, perineural invasion, and systemic inflammatory markers identifies a subgroup of patients with poor prognosis (Figure 5).

## 4. Discussion

Oral squamous cell carcinoma (OSCC) represents approximately 90% of all malignant tumors of the oral cavity and remains associated with significant morbidity and mortality, particularly in older populations and in male patients. In the present cohort, a clear male predominance and a higher incidence in the 61–70-year age group were observed, findings that are consistent with previously reported epidemiological data [4,5,6,7].

The present study provides additional insight into the prognostic relevance of systemic inflammatory markers in OSCC by evaluating their association with both survival outcomes and histopathological features. Unlike many previous studies that focused exclusively on the prognostic value of inflammatory biomarkers, the present study additionally evaluated their relationship with histopathological invasion patterns such as WPOI and perineural invasion. This combined approach may contribute to a better understanding of how systemic inflammation reflects local tumor aggressiveness in OSCC [5].

Histopathological parameters remain essential in determining tumor behavior. In our study, lymphovascular and perineural invasion did not show a statistically significant association with survival, despite being widely recognized as adverse prognostic factors in the literature. This discrepancy may be attributed to the relatively limited sample size and the retrospective nature of the study. Furthermore, recent evidence suggests that more detailed characterization of perineural invasion, such as the extent and diameter of invasion foci, may be necessary for accurate prognostic assessment [8,9,10,11,12].

In contrast, the worst pattern of invasion (WPOI) demonstrated a significant association with survival outcomes, with WPOI type 5 strongly correlated with increased mortality. This finding is in line with previous studies identifying WPOI as a reliable indicator of tumor aggressiveness and metastatic potential [12]. Additionally, our results suggest that tumor location may influence invasive behavior, as perineural and lymphovascular invasion were more frequently observed in tumors located in the tongue and floor of the mouth, which are known to be high-risk anatomical sites [13,14].

A central focus of this study was the evaluation of systemic inflammatory markers, specifically the neutrophil-to-lymphocyte ratio (NLR) and lymphocyte-to-monocyte ratio (LMR), as predictors of patient outcomes [15,16]. Our findings demonstrate that elevated NLR and decreased LMR values are significantly associated with poorer survival, supporting their role as prognostic indicators in OSCC. Notably, NLR emerged as an independent predictor of mortality in Cox regression analysis.

The cutoff value identified for NLR (2.88) showed moderate discriminative ability (AUC = 0.696) and is consistent with previously reported values in the literature. Several studies have demonstrated that an elevated NLR reflects a pro-inflammatory state associated with tumor progression, impaired immune surveillance, and reduced survival [15,16,17,18]. For example, Hasegawa et al. reported significantly worse survival outcomes in patients with NLR values greater than 3, findings that closely parallel those observed in our cohort [19]. Similarly, other authors have reported comparable threshold values, reinforcing the robustness of NLR as a prognostic biomarker [20,21].

The cutoff value identified for LMR (1.87) also demonstrated moderate predictive capacity (AUC = 0.664). Lower LMR values were associated with poorer outcomes, likely reflecting an increased monocyte population and subsequent differentiation into tumor-associated macrophages, which contribute to tumor progression and immune evasion [18]. Although LMR showed a protective trend in our analysis, its prognostic significance was not confirmed in multivariate models, suggesting that its impact may be influenced by additional clinical or pathological factors. Moreover, the Kaplan–Meier curves for LMR demonstrated partial overlap during follow-up, which may indicate a limitation of the proportional hazards assumption. This finding is likely related to the relatively small subgroup sizes and heterogeneity in patient outcomes over time. Therefore, the prognostic impact of LMR should be interpreted cautiously and requires validation in larger prospective cohorts.

The relationship between systemic inflammation and tumor biology is increasingly recognized as a key factor in cancer progression. Elevated NLR and reduced LMR reflect an imbalance between pro-tumor inflammatory processes and anti-tumor immune responses. These findings are further supported by studies demonstrating associations between inflammatory markers and tumor-related parameters such as depth of invasion (DOI) and WPOI, as well as the risk of metastasis [22,23,24,25,26,27].

A recent meta-analysis including 35 retrospective studies and 12,225 patients with OSCC further confirmed the prognostic significance of systemic inflammatory biomarkers, particularly NLR, while also comparing its predictive value with PLR and LMR (PMID: 41840623). The authors concluded that elevated NLR was consistently associated with poorer overall survival and disease progression, whereas the prognostic value of LMR appeared less robust across studies [28]. Our findings are consistent with these conclusions, as NLR emerged as an independent predictor of mortality, while LMR demonstrated only a non-significant protective trend in multivariate analysis. In contrast to large pooled analyses, the present study additionally explored the relationship between inflammatory markers and histopathological invasion patterns, particularly WPOI, thereby providing insight into the interaction between systemic inflammation and local tumor aggressiveness.

An important finding of our study is the prognostic relevance of integrating systemic inflammatory markers with adverse histopathological features into a combined risk stratification model. By incorporating NLR > 2.88, LMR < 1.87, perineural invasion, and WPOI 4–5 into a cumulative prognostic score, the study identified distinct survival differences among low-, intermediate-, and high-risk patient groups. The significantly poorer survival observed in the high-risk group underscores the additive prognostic value of combining tumor-related invasive characteristics with host inflammatory response parameters. In particular, elevated NLR emerged as an independent marker of increased mortality risk, supporting previous evidence that systemic inflammation promotes tumor progression, immune evasion, and aggressive biological behavior. Conversely, a higher LMR demonstrated a protective effect, suggesting that preserved lymphocyte-mediated immune surveillance may contribute to improved oncologic outcomes. Histopathological parameters such as perineural invasion and high-grade WPOI were also strongly associated with unfavorable prognosis, reflecting enhanced local invasiveness and tumor dissemination potential. The coexistence of these pathological features with systemic inflammatory imbalance appears to define a biologically aggressive subgroup of patients with markedly reduced survival. Therefore, this combined prognostic score may represent a practical and clinically applicable tool for identifying high-risk patients who could benefit from closer surveillance, intensified multimodal treatment strategies, and individualized therapeutic approaches.

This study has several limitations. Its retrospective design introduces potential selection and information biases, and the single-center setting may limit the generalizability of the findings. Additionally, the relatively small sample size may have reduced the statistical power to detect significant associations for certain variables. The absence of data on important risk factors, such as tobacco use, alcohol consumption, and HPV status, represents another limitation that may have influenced the observed outcomes.

Future prospective multicenter studies with larger cohorts are required to validate these findings and to establish standardized cutoff values for inflammatory markers. Further research should also explore the interaction between systemic inflammation, the tumor microenvironment, and treatment response to better define their role in personalized patient management.

## 5. Conclusions

Elevated NLR and reduced LMR were associated with poorer survival outcomes in patients with OSCC, with NLR demonstrating independent prognostic significance. WPOI type 5 was also associated with an unfavorable prognosis and appeared to correlate with more pronounced systemic inflammatory changes. These findings suggest that inflammatory biomarkers may complement histopathological assessment in OSCC. However, given the retrospective design and limited sample size, larger prospective studies are necessary to validate these observations.

By combining NLR, LMR, perineural invasion, and WPOI into a simple composite prognostic score, we identified distinct risk groups with significantly different survival outcomes. The novelty of this study lies in the development of an accessible and clinically applicable scoring model that incorporates both tumor microenvironment characteristics and host systemic inflammatory response, offering improved risk stratification beyond conventional pathological assessment alone.

## Figures and Tables

**Table 1:** Male-to-female ratio of patients diagnosed with oral squamous cell carcinoma.

Genre Prevalence					
		Frequency	Percent	Valid Percent	Cumulative Percent
Valid	F	29	18.5	18.5	18.5
	M	128	81.5	81.5	100.0
	Total	157	100.0	100.0	

**Table 2:** Age group distribution of patients diagnosed with oral squamous cell carcinoma.

Age Groups					
		Frequency	Percent	Valid Percent	Cumulative Percent
Valid	51–60	40	25.5	25.5	25.5
	61–70	55	35.0	35.0	60.5
	71–80	22	14.0	14.0	74.5
	over 80	14	8.9	8.9	83.4
	under 50	26	16.6	16.6	100.0
	Total	157	100.0	100.0	

**Table 3:** Vascular, perineural, and lymphatic invasion of squamous cell carcinoma in deceased and surviving patients.

Lympho-Vascular and Perineural Invasion								
*p*-0.53		Vascular Invasion = 1 Neural = 2 Lymphatic = 3 No Invasion = 4						Total
		1	1,2,3	1.2	1.3	2	4	
deceased = 1, surviving = 0	0	50.0%	37.5%	16.7%	33.3%	40.0%	51.4%	46.5%
	1	50.0%	62.5%	83.3%	66.7%	60.0%	48.6%	53.5%
Total		100.0%	100.0%	100.0%	100.0%	100.0%	100.0%	100.0%

**Table 4:** WPOI in surviving and deceased patients diagnosed with oral SCC.

WPOI vs. Negative Outcome										
*p*-0.003			WPOI							Total
				Type 1	Type 2	Type 3	Type 4	Type 5	Type 1–4	
deceased = 1, surviving = 0	0	No:	37	1	0	2	5	7	21	73
		% WPOI	40.7%	100.0%	0.0%	25.0%	100.0%	36.8%	65.6%	46.5%
	1	No:	54	0	1	6	0	12	11	84
		% WPOI	59.3%	0.0%	100.0%	75.0%	0.0%	63.2%	34.4%	53.5%
Total		No:	91	1	1	8	5	19	32	157
		% WPOI	100.0%	100.0%	100.0%	100.0%	100.0%	100.0%	100.0%	100.0%

**Table 5:** (**a**) Mann–Whitney test for non-parametric data. (**b**) The prognostic role of N/L and L/M ratios in the negative outcome of patients diagnosed with oral squamous cell carcinoma.

(**a**)			
**ROC Curve NLR and LMR**			
Deceased = 1, surviving = 0		ratio N/L	ratio L/M
0	Mean	3.779071	3.467276
	Std. Deviation	3.5421490	2.8408857
	Median	2.751055	2.805361
	Minimum	0.7882	0.4256
	Maximum	20.6636	22.0896
1	Mean	14.735429	2.877414
	Std. Deviation	54.9760288	5.5101187
	Median	3.996951	1.978776
	Minimum	0.1158	0.2857
	Maximum	495.0000	51.0000
Total	Mean	9.641072	3.149658
	Std. Deviation	40.5450108	4.4768227
	Median	3.700000	2.414790
	Minimum	0.1158	0.2857
	Maximum	495.0000	51.0000
(**b**)			
		ratio N/L	ratio L/M
Mann–Whitney U		1866.500	2029.500
Wilcoxon W		4567.500	5599.500
Z		−4.221	−3.535
*p* value		0.0001	0.0001
Grouping Variable: deceased = 1, surviving = 0			

## Data Availability

The data presented in this study are available upon request from the corresponding author.

## Abbreviations

The following abbreviations are used in this manuscript:

OSCC	Oral squamous cell carcinoma
NLR	Neutrophile-to-lymphocyte ratio
LMR	Lymphocyte-to-monocyte ratio
DOI	Depth of invasion
WPOI	Worst pattern of invasion
ROC	Receiver operating characteristic
